# Treatment Strategies in Emergency Endoscopy for Acute Esophageal Variceal Bleeding (CHESS1905): A Nationwide Cohort Study

**DOI:** 10.3389/fmed.2022.872881

**Published:** 2022-04-27

**Authors:** Yifei Huang, Wenhui Zhang, Huiling Xiang, Yanna Liu, Lili Yuan, Liyao Zhang, Shengjuan Hu, Dongli Xia, Jia Li, Min Gao, Xing Wang, Xingsi Qi, Lijun Peng, Ying Song, Xiqiao Zhou, Jing Zeng, Xiaoyan Tan, Mingming Deng, Haiming Fang, Shenglin Qi, Song He, Yongfeng He, Bin Ye, Wei Wu, Tong Dang, Jiangbo Shao, Wei Wei, Jianping Hu, Xin Yong, Chaohui He, Jinlun Bao, Yuening Zhang, Guo Zhang, Rui Ji, Yang Bo, Wei Yan, Hongjiang Li, Yanling Wang, Mengmeng Li, Fengmei Wang, Jia Lian, Chang’en Liu, Ping Cao, Zhenbei Liu, Aimin Liu, Lili Zhao, Shuang Li, Yunhai Wu, Ye Gu, Yan Wang, Yanfei Fang, Pan Jiang, Bin Wu, Chuan Liu, Xiaolong Qi

**Affiliations:** ^1^Institute of Portal Hypertension, The First Hospital of Lanzhou University, Lanzhou, China; ^2^Beijing Shijitan Hospital, Beijing, China; ^3^Diagnosis and Treatment Center, The Fifth Medical Center of PLA General Hospital, Beijing, China; ^4^Department of Hepatology and Gastroenterology, Tianjin Third Central Hospital, Tianjin, China; ^5^Department of Microbiology and Infectious Disease Center, School of Basic Medical Sciences, Peking University Health Science Center, Beijing, China; ^6^Department of Gastroenterology, Shanxi Bethune Hospital, Taiyuan, China; ^7^Department of Critical Care Medicine, The Sixth People’s Hospital of Shenyang, Shenyang, China; ^8^Department of Gastroenterology, Endoscopic Center, People’s Hospital of Ningxia Hui Autonomous Region, Yinchuan, China; ^9^Department of Gastroenterology, Chongqing Fuling Central Hospital, Chongqing, China; ^10^Department of Gastroenterology and Hepatology, Tianjin Second People’s Hospital, Tianjin, China; ^11^Department of Gastroenterology, Sir Run Run Shaw Hospital, School of Medicine, Zhejiang University, Hangzhou, China; ^12^Department of Gastroenterology, The Third Affiliated Hospital of Sun Yat-sen University, Guangzhou, China; ^13^Department of Gastroenterology, The Affiliated Hospital of Qingdao University, Qingdao, China; ^14^Department of Gastroenterology, Linyi People’s Hospital, Linyi, China; ^15^Department of Gastroenterology, Xi’an GaoXin Hospital, Xi’an, China; ^16^Department of Gastroenterology, First Affiliated Hospital of Nanjing Medical University, Nanjing, China; ^17^Department of Emergency, Huizhou Third People’s Hospital, Guangzhou Medical University, Huizhou, China; ^18^Department of Gastroenterology, Maoming People’s Hospital, Maoming, China; ^19^Department of Gastroenterology, The Affiliated Hospital of Southwest Medical University, Luzhou, China; ^20^Department of Gastroenterology and Hepatology, The Second Hospital of Anhui Medical University, Hefei, China; ^21^Department of Hepatology, Dalian Sixth People’s Hospital, Dalian, China; ^22^Department of Gastroenterology, The Second Affiliated Hospital of Chongqing Medical University, Chongqing, China; ^23^Department of Gastroenterology, Endoscopic Center, Ankang Central Hospital, Ankang, China; ^24^Department of Gastroenterology, Lishui Hospital of Zhejiang University, The Fifth Affiliated Hospital of Wenzhou Medical University, Lishui Central Hospital, Lishui, China; ^25^Department of Gastroenterology, The First Affiliated Hospital of Wenzhou Medical University, Wenzhou, China; ^26^Inner Mongolia Institute of Digestive Diseases, The Second Affiliated Hospital of Baotou Medical College, Inner Mongolia University of Science and Technology, Baotou, China; ^27^Department of Liver Disease, The Third People’s Hospital of Zhenjiang, Zhenjiang, China; ^28^Department of Gastroenterology, Jinhua Hospital, Jinhua, China; ^29^Department of Gastroenterology, First People’s Hospital of Yinchuan City, Yinchuan, China; ^30^Gastroenterology, General Hospital of Western Theater Command, Chengdu, China; ^31^Department of Gastroenterology and Endoscopy, The Fifth Affiliated Zhuhai Hospital of Zunyi Medical University, Zhuhai, China; ^32^Department of Gastroenterology, Shannan People’s Hospital, Shannan, China; ^33^Center of Hepatology and Gastroenterology, Beijing You’an Hospital, Capital Medical University, Beijing, China; ^34^The People’s Hospital of Guangxi Zhuang Autonomous Region, Nanning, China; ^35^Department of Gastroenterology, The First Hospital of Lanzhou University, Lanzhou, China; ^36^Department of Hepatobiliary Surgery, People’s Hospital of Ningxia Hui Autonomous Region, Yinchuan, China; ^37^Department of Gastroenterology, Tongji Hospital, Tongji Medical College, Huazhong University of Science and Technology, Wuhan, China; ^38^Department of Hepatology, Baoding People’s Hospital, Baoding, China

**Keywords:** liver cirrhosis, portal hypertension, emergency endoscopy, endoscopic injection sclerotherapy, endoscopic variceal ligation

## Abstract

**Background and Aims:**

Emergency endoscopy is recommended for patients with acute esophageal variceal bleeding (EVB) and their prognosis has improved markedly over past decades due to the increased specialization of endoscopic practice. The study aimed to compare outcomes following emergency endoscopic injection sclerotherapy (EIS) and endoscopic variceal ligation (EVL) in cirrhotic patients with acute EVB.

**Methods:**

Cirrhotic patients with acute EVB who underwent emergency endoscopy were retrospectively enrolled from 2013 to 2020 across 34 university hospitals from 30 cities. The primary outcome was the incidence of 5-day rebleeding after emergency endoscopy. Subgroup analysis was stratified by Child-Pugh class and bleeding history. A 1:1 propensity score matching (PSM) analysis was performed.

**Results:**

A total of 1,017 and 382 patients were included in EIS group and EVL group, respectively. The 5-day rebleeding incidence was similar between EIS group and EVL group (4% vs. 5%, *P* = 0.45). The result remained the same after PSM (*P* = 1.00). Among Child-Pugh class A, B and C patients, there were no differences in the 5-day rebleeding incidence between the two groups after PSM (*P* = 0.25, 0.82, and 0.21, respectively). As for the patients with or without bleeding history, the differences between EIS group and EVL group were not significant after PSM (*P* = 1.00 and 0.26, respectively).

**Conclusion:**

The nationwide cohort study indicates that EIS and EVL are both efficient emergency endoscopic treatment strategies for acute EVB. EIS should not be dismissed as an economical and effective emergency endoscopic treatment strategy of acute EVB. ClincialTrials.gov number NCT04307264.

## Introduction

Portal hypertension is one of the most important factors affecting the clinical course of patients with cirrhosis, as it can predict the development of cirrhosis-related complications, such as esophageal variceal bleeding (EVB), a potentially lethal manifestation of cirrhosis and portal hypertension (1). The lifetime prevalence of esophageal varices (EV) in subjects with cirrhosis and portal hypertension ranges between 60 and 80%, with acute EVB being the most serious complication which occurs in 1/3 of patients with EV (2, 3). Standardization of supportive care and new therapeutic options reduced bleeding-related mortality from about 50 to 15 –20% in the last three decades (4).

International consensus recommends emergency endoscopy within 24 h after gastroenterologic consultation for patients with acute upper gastrointestinal bleeding (5). Emergency endoscopy allows timely identification as well as treatment of bleeding, which reduces the risk of early rebleeding and death and the need for surgery (6). Endoscopic injection sclerotherapy (EIS) and endoscopic variceal ligation (EVL) are favored non-surgical treatment strategies of endoscopy for managing acute EVB (7–10). A newly published meta-analysis has demonstrated the superiority of EVL over EIS in terms of fewer adverse events (11). Several trials comparing long-term EIS to EVL gave separate data for EVB, however, failing to show any differences between the two (12, 13). Only a few previous trials with small sample sizes have been specifically addressed to compare these emergency endoscopic treatment strategies in acute variceal bleeding and results were contradictory (14–17). Therefore, this lack of consistency among studies raises the question regarding the superiority of treatment strategies of emergency endoscopy in patients with acute EVB.

This study aims to assess the incidence of early rebleeding, in-hospital mortality, need for intensive care unit (ICU), and the length of hospital stay of cirrhotic patients with acute EVB receiving emergency EIS or EVL.

## Materials and Methods

### Study Design and Patients

This study was conducted using the database of a multi-center, observational study (CHESS1905, ClinicalTrials.gov identifier: NCT04957875) to evaluate the optimal endoscopy timing for acute variceal bleeding in patients with cirrhosis. Patients were enrolled from 34 university hospitals from 30 cities in China between February 2013 and May 2020. Two independent investigators (Liu C and Huang Y) reviewed the medical records, including demographic, laboratory, and endoscopic data.

Inclusion criteria were as follows: (1) age ≥18 years; (2) established diagnosis of cirrhosis (based on liver biopsy or the combination of clinical, biochemical, and imaging findings); (3) witnessed or reported evidence of gastrointestinal hemorrhage (hematemesis, melena, or hematochezia); (4) endoscopy confirmed EV as the only source of bleeding; and (5) had emergency EIS or EVL alone. Exclusion criteria were as follows: (1) gastric variceal bleeding; (2) severe heart failure, chronic obstructive pulmonary disease, and malignancy; (3) previous EIS or EVL within 3 months; (4) incomplete or missing data. This study was approved by the Institutional Ethics Committee of included hospitals. Written informed consent was obtained from all the patients for their data by respective hospital investigators to be used for research purposes.

### Treatment

When cirrhotic patients presented with acute EVB to the emergency department, emergency physicians consulted gastroenterologists on duty to assess the patient for suitability for endoscopy, usually after initial stabilization. Therapeutic endoscopy for acute EVB was performed within 24 h after consultation by an experienced attending endoscopist. Written informed consent for endoscopy was obtained before each procedure. The standard of care at all hospitals was to administer a vasoactive agent and antibiotics upon the patient’s presentation. Packed red blood cells were transfused at the discretion of the attending gastroenterologist.

Emergency endoscopic procedures were performed by experienced endoscopists using conventional standard forward-viewing upper gastrointestinal video endoscopes at individual centers. Treatment strategies were determined by endoscopists due to experience.

Endoscopic injection sclerotherapy: An intravariceal or a paravariceal injection of 10–30 mL of lauromacrogol (Tianyu Pharmaceutical, Shanxi, China) was administered through the injection needle. The initial injections were administered 2–3 cm above the gastroesophageal junction and continued until all EV were treated.

Endoscopic variceal ligation: Six to Twelve multiband ligators were used. The ligations were initiated 1 cm above the gastroesophageal junction and proceeded to the next proximal varix. No more than 14 bands were positioned per session.

### Outcomes

The primary outcome was the incidence of 5-day rebleeding after emergency EIS or EVL. The secondary outcomes included the in-hospital mortality, need for ICU, and the length of hospital stay. Rebleeding was defined as new-onset hematemesis, coffee ground vomiting, melena, or hematochezia with accompanying laboratory abnormality consistent with bleeding (specifically, a drop in hemoglobin of greater than 2 g/dL within 24 h) or vital sign changes (systolic blood pressure [SBP] decreasing to <90 mmHg or heart rate increasing to >100 beats/min).

### Propensity Score Matching

Patients in the EIS group were matched to the EVL group using the closest estimated propensity score within 0.1 of the standard deviation of the logit of propensity score matching (PSM) to achieve a balance at baseline (i.e., minimal confounding). Final covariates included the following variables: SBP <90 mmHg, heart rate >100 beats/min, bleeding history, hemoglobin, aspartate aminotransferase (AST), and prothrombin time (PT).

### Statistical Analysis

We calculated that in order to prove a difference of 6%-point difference (6% vs. 12%) in the primary outcome between the groups (emergency EIS and emergency EVL) with a power of 80% and a significance level lower than 5%, each group should contain at least 330 patients.

Continuous variables were reported as median with interquartile range (IQR) or mean with standard deviation (SD), and were compared using the Mann-Whitney test or the Student’s *t*-test. Categorical data, presented as number and frequencies (%), were compared using the Chi-square test, or the Fisher’s exact test. The cumulative probability curves were generated using the Kaplan–Meier method and compared using the log-rank test. Univariate and multivariate analyses were assessed using a Cox proportional hazards stepwise model. Factors with a *P* < 0.05 on univariate analysis were incorporated into multivariate analysis. After PSM, univariate, multivariate logistic regression and Kaplan–Meier analyses were also performed. The data analyses were performed using the R language [Version 4.0.3, R Core Team (18)]. A *P* < 0.05 was considered statistically significant.

## Results

### Patient Characteristics

**FIGURE 1:**
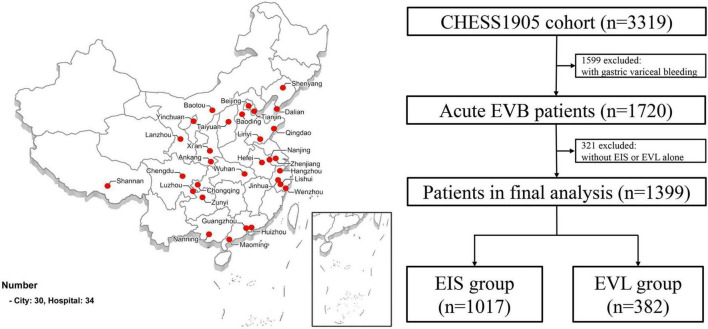
Study flow chart. EV, esophageal varices; EVB, esophageal variceal bleeding; EIS, endoscopic injection sclerotherapy; EVL, endoscopic variceal ligation.

The study flow chart was shown in Figure 1 and baseline characteristics of the enrolled patients were summarized in Table 1. A total of 1,017 and 382 patients were included in the emergency EIS group and the emergency EVL group, respectively. Hepatitis B, alcohol-related liver disease, and hepatitis C were the most three common etiologies of cirrhosis in 582 (57%), 203 (20%) and 100 (10%) patients in the emergency EIS group, and 209 (55%), 61 (16%), and 36 (9%) patients in the emergency EVL group, respectively. The groups were similar in terms of age, gender, Child Pugh class, platelet count (PLT), and alanine aminotransferase (ALT). SBP <90 mmHg, heart rate >100 beats/min, history of EVB, hemoglobin, AST, and PT were significantly different between the two groups (*P* < 0.05). After PSM, all features became well-balanced and there were 369 patients in each of the EIS group and the EVL group.

**TABLE 1:** Baseline characteristics of patients.

Variables	Before PSM			After PSM		
	EIS group (*n* = 1017)	EVL group (*n* = 382)	*P-*value	EIS group (*n* = 369)	EVL group (*n* = 369)	*P-*value
Age (year), mean (SD)	54.93 (11.40)	56.06 (11.85)	0.10	56.43 (10.80)	56.10 (11.81)	0.69
Male, n (%)	695 (68%)	257 (67%)	0.70	239 (65%)	249 (67%)	0.44
SBP <90 mmHg, n (%)	47 (5%)	34 (9%)	<0.05	25 (7%)	30 (8%)	0.48
HR >100 beats/min, n (%)	154 (15%)	93 (24%)	<0.05	86 (23%)	88 (24%)	0.86
With a bleeding history, n (%)	653 (64%)	198 (52%)	<0.05	170(46%)	166 (45%)	0.77
With gastric varices, n (%)	677 (66.57%)	69 (18.06%)	<0.05	75 (20.33%)	67 (18.16%)	0.22
Child-Pugh class, n (%)			0.09			0.32
Class A	279 (27%)	131 (34%)		140 (38%)	131 (36%)	-
Class B	586 (58%)	193 (51%)		184 (50%)	184 (50%)	-
Class C	152 (15%)	58 (15%)		45 (12%)	54 (15%)	-
**Laboratory tests, median (IQR)**						
Hemoglobin (g/L)	76.02 (29.08)	81.82 (25.01)	<0.05	82.88 (24.18)	82.01 (25.05)	0.63
PLT (10^9^/L)	74.04 (59.00)	85.92 (57.00)	0.10	73.00 (51.00)	77.00 (57.00)	0.84
AST (U/L)	24.08 (20.09)	28.00 (24.70)	<0.05	24.00 (17.25)	28.00 (25.00)	0.28
ALT (U/L)	34.00 (29.13)	33.39 (34.00)	0.09	33.00 (27.00)	33.09 (34.07)	0.29
PT (s)	14.42 (3.45)	15.65 (3.83)	<0.05	14.50 (3.43)	15.60 (3.65)	0.18

*PSM, propensity score matching; EIS, endoscopic injection sclerotherapy; EVL, endoscopic variceal ligation; SD, standard deviation; SBP, systolic blood pressure; HR, heart rate; IQR, interquartile range; PLT, platelet count; AST, aspartate aminotransferase; ALT, alanine aminotransferase; PT, prothrombin time.*

### Five-Day Rebleeding and Prognostic Indicators

**FIGURE 2:**
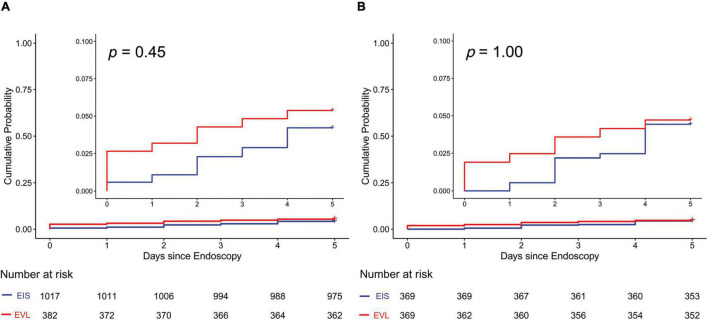
Cumulative probability of 5-day rebleeding in all patients with cirrhosis. **(A)** Before PSM; **(B)** after PSM. EIS, endoscopic injection sclerotherapy; EVL, endoscopic variceal ligation; PSM, propensity score matching.

Before PSM, the overall 5-day rebleeding rate was 4% (*n* = 62). The difference in the incidence of rebleeding between the EIS and the EVL groups was not statistically significantly different (4% vs. 5%, *P* = 0.45) (Figure 2A and Table 2). After PSM, the overall 5-day rebleeding rate was 4% (*n* = 33). Differences between the two groups remained insignificant (4% vs. 4%, *P* = 1.00) (Figure 2B and Table 2).

**TABLE 2:** Outcomes in the two groups.

Outcome	Before PSM			After PSM		
	EIS group (*n* = 1,017)	EVL group (*n* = 382)	*P-*value	EIS group (*n* = 369)	EVL group (*n* = 369)	*P-*value
**Primary outcome**						
Rebleeding, n (%)	42 (4%)	20 (5%)	0.45	16 (4%)	17 (4%)	1.00
**Secondary outcome**						
Death, n (%)	10 (1%)	5 (1%)	1.00	2 (1%)	4 (1%)	0.41
Need for ICU, n (%)	204 (20%)	82 (21%)	0.79	61 (17%)	74 (20%)	0.21
Length of hospital stay, mean (SD)	17.24 (11.03)	11.94 (7.36)	<0.05	16.98 (10.55)	11.89 (7.28)	<0.05

*PSM, propensity score matching; EIS, endoscopic injection sclerotherapy; EVL, endoscopic variceal ligation; ICU, intensive care unit; SD, standard deviation.*

Univariate analyses of all patients before PSM demonstrated that SBP <90 mmHg, Child-Pugh class, and PLT were independently related to cumulative probability of 5-day rebleeding. Further Cox multivariate analysis showed that the three factors were associated with 5-day rebleeding (Table 3). After PSM, univariate analyses in all patients demonstrated that Child-Pugh class, hemoglobin, AST, and ALT were the independent risk factor related to 5-day rebleeding (Table 3). On multivariable analysis, Child-Pugh class (hazard ratio [HR], 1.82; 95% confidence intervals [CI], 1.09-3.05) and hemoglobin were associated with early rebleeding (HR, 0.99; 95% CI, 0.97-1.00) (Table 3).

**TABLE 3:** Univariate and multivariate analysis of 5-day re-bleeding.

Predictor variables	Before PSM				After PSM			
	Univariate analysis		Multivariate analysis		Univariate analysis		Multivariate analysis	
	HR (95% CI)	*p-*value	HR (95% CI)	*P-*value	HR (95% CI)	*p-*value	HR (95% CI)	*P*-value
Age	1.01 (0.99–1.04)	0.23			1.01 (0.98–1.04)	0.55		
Male	1.56 (0.93–3.00)	0.17			1.62 (0.73–3.57)	0.23		
SBP <90 mmHg	0.13 (0.02–0.98)	<0.05	0.14 (0.02 – 1.10)	<0.05	0.39 (0.05–2.80)	0.34		
HR >100 beats/min	1.55 (0.80–3.03)	0.20			1.42 (0.68–2.98)	0.35		
With a bleeding history	1.05 (0.62–1.80)	0.85			1.12 (0.57–2.21)	0.75		
Child-Pugh class	1.75 (1.15–2.65)	<0.05	1.82 (1.23 – 2.71)	<0.05	2.02 (1.23–3.32)	<0.05	1.82 (1.09–3.05)	<0.05
Hemoglobin (g/L)	1.00 (0.98–1.01)	0.42			0.98 (0.97–1.00)	<0.05	0.99 (0.97–1.00)	<0.05
Platelet count (10^9^/L)	1.00 (1.00–1.00)	<0.05	1.00 (1.00 – 1.00)	<0.05	1.00 (1.00–1.01)	0.32		
AST (U/L)	1.00 (0.99–1.00)	0.57			1.00 (1.00–1.00)	<0.05	1.00 (1.00–1.00)	0.76
ALT (U/L)	1.00 (1.00–1.00)	0.47			1.00 (1.00–1.00)	<0.05	1.00 (0.99–1.01)	0.61
PT (s), median (IQR)	1.00 (0.97–1.03)	0.85			1.01 (0.99–1.03)	0.45		

*PSM, propensity score matching; HR, hazard ratio; CI, confidence intervals; SBP, systolic blood pressure; HR, heart rate; AST, aspartate aminotransferase; ALT, alanine aminotransferase; PT, prothrombin time; IQR, interquartile range.*

### Five-Day Rebleeding Stratified by Child-Pugh Class and bleeding History

Among Child-Pugh class A, B and C patients, the differences in the 5-day rebleeding incidence were all not significant between EIS and EVL groups (before PSM, 3% vs. 2%, *P* = 0.83; 4% vs. 6%, *P* = 0.31; 7% vs. 9%, *P* = 0.96, respectively; after PSM, 0% vs. 2%, *P* = 0.25; 6% vs. 5%, *P* = 0.82; 2% vs. 9%, *P* = 0.21, respectively) (Supplementary Figure 1).

Among the patients with a first episode of bleeding or the patients with a previous episode of bleeding, the differences of the 5-day rebleeding incidences between EIS and EVL group were not significant (before PSM, 3% vs. 5% *P* = 0.40; 5% vs. 5%, *P* = 0.85; after PSM, 1% vs. 4% *P* = 1.00; 2% vs. 4%, *P* = 0.20) (Supplementary Figure 2).

### In-Hospital Mortality, Need for Intensive Care Unit and the Length of Hospital Stay

Overall, there were 15 (1%) in-hospital deaths. The in-hospital mortality in the emergency EIS group and the emergency EVL group were similar (1% vs. 1%, *P* = 1.00) (Table 2). After PSM, the in-hospital mortality between two groups remained similar (1% vs. 1%, *P* = 0.41) (Table 2).

The overall number of patients who required ICU care was 732 (22.1%). The differences in the need for ICU care in the emergency EIS group and the emergency EVL group were similar (before PSM, 20% vs. 21%, *P* = 0.79; after PSM, 17% vs. 20%, *P* = 0.21) (Table 2).

The mean length of hospital stay was significantly higher in the emergency EIS group (17.24 days) than that in the EVL group (11.94 days) (*P* < 0.05) (Table 2). After PSM, the mean length of hospital stay in the emergency EIS group remained longer (16.98 days vs. 11.89 days, *P* < 0.05) (Table 2).

## Discussion

The present study, to our best knowledge, is the largest to report on the appropriate endoscopic strategy for acute EVB. After PSM analysis, the overall results indicated that choice of treatment strategy of emergency endoscopy (EIS or EVL) made no difference in the incidence of rebleeding within 5 days among cirrhotic patients with acute EVB.

Although the superiority of EVL over EIS for the secondary prophylaxis of variceal hemorrhage has been proven based on moderate-certainty evidence (11), the better emergency endoscopic treatment for acute EVB remains controversial. Lo GH, et al. compared the short-term efficacy and safety of emergency EIS with EVL in the arresting of acute EVB. Their results demonstrated that EVL was superior to EIS in the control of spurting varices and patients treated with EVL required fewer vasoconstrictors and fewer transfusion units than patients treated with EIS (14). A comparison of EIS with EVL for the emergency endoscopic treatment of acute variceal bleeding was conducted in 2006 in 179 patients (89 in the EIS group and 90 in the EVL group). Treatment failure occurred in 24% of EIS patients and in 10% of EVL patients (relative risk: 2.4%). The major adverse effect rate was found to be 13% for those receiving endoscopic EIS and 4% for those in the EVL group (*P* = 0.04) (15). On the contrary, one study suggested that emergency EIS might be more effective (16). In addition, Luz et al. (17) reported that no differences were found between EIS group (*n* = 50) and EVL group (*n* = 50) for bleeding control, early re-bleeding rates, complications, or mortality. Similar to this literature, the present study showed no difference between emergency EIS and EVL group for 5-day rebleeding rate (4% vs. 5%, *P* = 0.45); and the result remained unchanged after PSM analysis (4% vs. 4%, *P* = 1.00).

High-risk factors for rebleeding and mortality of patients with acute EVB have been reported. Hsu et al. suggested that hematemesis, delayed endoscopy (>15 h), first failure of hemostasis and high MELD score are independent risk factors for in-hospital mortality (19). Monescillo et al. reported that the platelet-albumin-bilirubin score at baseline was an indicator for early rebleeding and mortality of patients with acute variceal bleeding (20).

Moreover, the severity of the underlying liver disease could be predictive parameter of prognosis and furthermore helped with risk stratification for patients with acute EVB, individualizing treatment strategies (21). The present results demonstrated that Child-Pugh class and hemoglobin were associated with early rebleeding. In subgroup analysis, different Child-Pugh class didn’t affect the difference in 5-day rebleeding incidence between emergency EIS and EVL groups.

Our study additionally revealed that no differences were found between EIS group and EVL groups for in-hospital mortality (1% vs. 1%, *P* = 0.41) and need for ICU (17% vs. 20%, *P* = 0.21), both before and after PSM. The mean length of hospital stay was significantly higher in the emergency EIS group than that in the emergency EVL group after PSM (16.98 days vs. 11.89 days, *P* < 0.05). Possible explanation for this difference might be fewer complications associated with EVL than EIS (ulcer, stenosis and even perforation) (22–25). However, despite fewer complications of EVL involving the esophageal wall, the hemorrhage is lethal when bands fall off.

Based on the above results, we suggest that EIS is comparable with EVL in the emergency endoscopic treatment for acute EVB. EIS and EVL were both feasible treatment therapies of emergency endoscopy for hemostasis and preventing rebleeding (7, 8). EIS was introduced into clinical practice 50 years before EVL (26). Unlike EVL, there were many variates in EIS, such as the type and concentration of sclerosing agents, injected volume and location and so on. Meanwhile, EIS was a more operator-dependent technique than EVL. This operation needed experienced endoscopist with significant skills. EVL was a relatively novel technique described by Stiegmann et al. (27). In contrast to EIS, EVL was acted by mechanical action and was easy to perform with generally reproducible and homogeneous results (28, 29). Triantos et al. suggested that sclerosing agents could be injected through the endoscopy immediately after diagnosis of acute EVB, while for EVL, it is necessary to withdraw the endoscope for system assembly, thus increasing procedural time and complication risk (27). Furthermore, the economic perspective on the emergency endoscopic treatment of acute EVB should be emphasized. A ligation device with six elastic bands currently costs ¥2500.00 on average. In comparison, a sclerosis needle costs approximately ¥400.00, and the sclerosing substance costs approximately ¥1200.00. The use of less costly but similarly efficient technique is a sensible choice (30).

Several limitations of the study were notable. Firstly, data from this nationwide cohort were acquired retrospectively, and therefore could lead to selection bias. Although this weakness was likely offset by the implementation of rigorous methodology to identify patients with acute EVB and define bleeding, and by the inclusion of a substantial sample size, prospective studies should be conducted to further evaluate the impact of urgent endoscopy on patients with acute variceal bleeding. Meanwhile, we were lack of data of complications and costs of enrolled patients, the comparison of complications between emergency EVL and EIS groups and cost-effective study should be explored in the future. Secondly, most of our patients had a background of hepatitis B virus-related cirrhosis, which reflected the current real-world situation in many parts of Asia-Pacific regions. Whether the results obtained in this study can be extrapolated to hepatitis C virus, alcohol or non-alcoholic steatohepatitis-related cirrhosis is not certain. Lastly, recent Baveno VII consensus recommended that six-week mortality should be the primary endpoint for studies on the treatment of acute EVB (31). However, given the design of the study, we could only define the incidence of rebleeding within 5 days, in-hospital mortality, need for ICU and the length of hospital stay as outcomes. Long-term follow-up and overall survival should be identified further.

## Conclusion

The nationwide cohort study indicates that EIS and EVL are both efficient emergency endoscopic treatment strategies for acute EVB. EIS should not be dismissed as an economical and effective emergency endoscopic treatment strategy of acute EVB.

## Data Availability Statement

The raw data supporting the conclusions of this article will be made available by the authors, without undue reservation.

## Ethics Statement

The studies involving human participants were reviewed and approved by Clinical Research Ethics Committee, First Hospital of Lanzhou University. The patients/participants provided their written informed consent to participate in this study.

## Author Contributions

XLQ: study concept, design, and critical revision of the manuscript. WZ, HX, YL, LY, DX, JLi, LYZ, MG, XW, XSQ, LP, YS, XZ, JZ, XT, MD, HF, SQ, SH, YiH, BY, WWu, TD, JS, WWe, JH, XY, CH, JB, YZ, GZ, RJ, YB, SJH, WY, HL, YLW, ML, FW, JLia, CaL, PC, ZL, AL, LLZ, SL, YHW, YG, YW, YF, PJ, and BW: acquisition of data and technique support. YoH and CuL: analysis and interpretation of data. YiH: drafting of the manuscript. All authors read and approved the final manuscript.

## Conflict of Interest

The authors declare that the research was conducted in the absence of any commercial or financial relationships that could be construed as a potential conflict of interest.

## Publisher’s Note

All claims expressed in this article are solely those of the authors and do not necessarily represent those of their affiliated organizations, or those of the publisher, the editors and the reviewers. Any product that may be evaluated in this article, or claim that may be made by its manufacturer, is not guaranteed or endorsed by the publisher.
