# Supplementary material for: Treatment Strategies in Emergency Endoscopy for Acute Esophageal Variceal Bleeding (CHESS1905): A Nationwide Cohort Study
Source: Front Med (Lausanne). 2022 Apr 27;9:872881. doi: 10.3389/fmed.2022.872881 (PMC9092278; doi:10.3389/fmed.2022.872881)
Supplement: Supplementary file 1 [file Data_Sheet_1.pdf]

Supplementary Material

Supplementary Figures

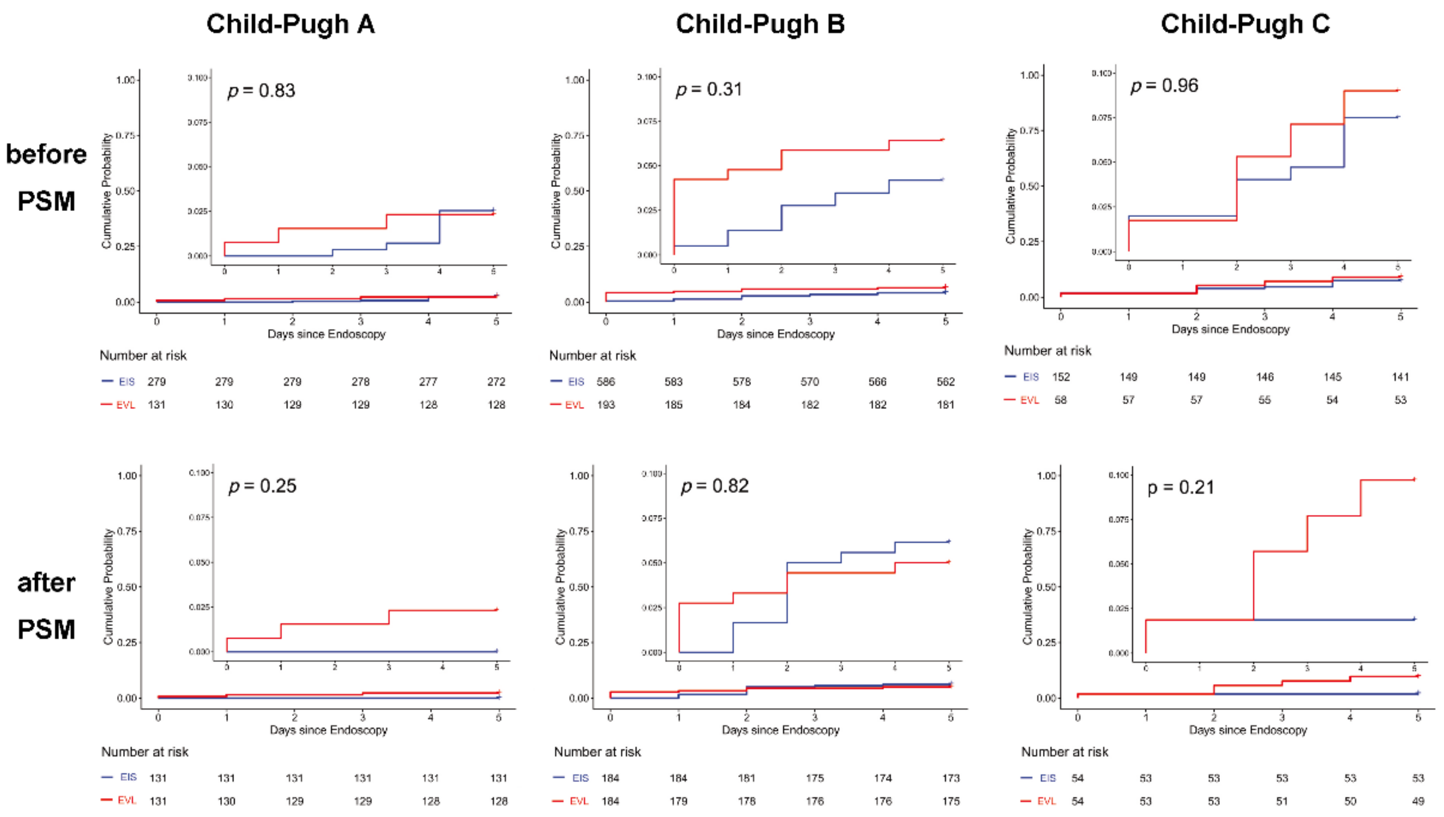

Supplementary Figure 1. Cumulative probability of 5-day rebleeding in Child-Pugh A, B or C patients with cirrhosis.

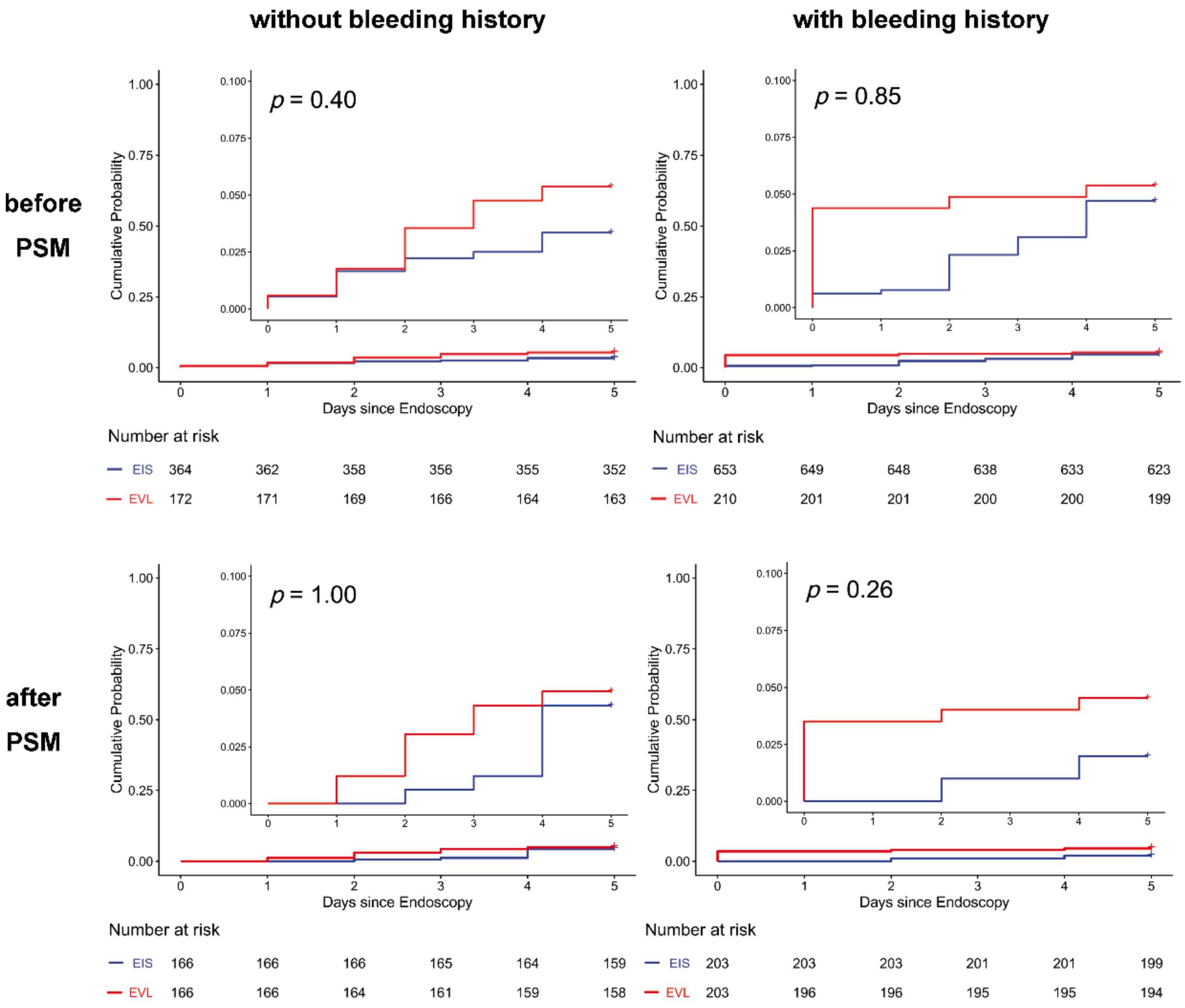

Supplementary Figure 2. Cumulative probability of 5-day rebleeding in cirrhotic patients with or without a bleeding history.
